# Empowerment or Pressure? Exploring the Impact of Female Body Depictions in Body Positivity Instagram Posts on Self‐Objectification

**DOI:** 10.1002/ijop.70208

**Published:** 2026-04-15

**Authors:** Nina Krizova, Nikol Kvardova, Hana Machackova

**Affiliations:** ^1^ Interdisciplinary Research Team on Internet and Society, Faculty of Social Studies Masaryk University Brno Czech Republic

**Keywords:** attitudes towards body positivity, body image, body positivity posts, female body portrayals, state self‐objectification

## Abstract

Although Body Positivity content (BoPo) has been criticised for emphasising physical appearance and promoting self‐objectification, the specific features driving these effects remain unclear. The present study examined whether depictions of female bodies act as triggers for self‐objectification in BoPo on Instagram. In a between‐subjects online experiment involving 158 women aged 18–29 (M = 21.6, SD = 2.4), exposure to female bodies in BoPo posts did not heighten state self‐objectification. Trait self‐objectification and negative mood did not moderate these effects; however, women with negative attitudes towards BoPo reported higher levels of state self‐objectification. These findings underscore the potential importance of subjective appraisals in shaping the impacts of BoPo content. Overall, the study contributes to the ongoing debate about the potentially negative effects of BoPo on Instagram, suggesting that body depictions alone may not reinforce self‐objectification. Future research should examine the distinct influence of different types of body portrayals to further clarify the impact of BoPo content on body image. From a practical perspective, prevention efforts and social media campaigns should aim to raise awareness of BoPo features that continue to overemphasise appearance, helping women better protect their body image from potential adverse effects.

## 1 Introduction

Contemporary society emphasises the importance of physical appearance. Media constantly portray unattainable thin and sexually appealing female bodies (Vendemia et al. 2021), leading to 85% of women desiring to change their appearance (Vandervoort et al. 2015). To counter this, social media respond with content that promotes Body Positivity (BoPo), which encompasses posts highlighting the appreciation of all body types, the importance of ‘inner beauty’, the functionality above appearance, the need for self‐care regardless of weight or height, and protective filtering of body‐related information (Cohen, Irwin, et al. 2019). BoPo challenges mainstream beauty ideals and the unrealistic nature of media appearance imagery (e.g., through portraying photos of real, unposed, and unmodified appearance; Cohen, Irwin, et al. 2019) and displays captions like ‘everybody is beautiful’ or ‘your value is not determined by your weight’. By doing so, BoPo strives to alleviate appearance pressures and encourage positive body image.

Despite the body image‐promoting goals, BoPo has been criticised for continuing to feature contradictory messages, such as thin praise alongside weight stigmatisation and weight loss promotion (Lazuka et al. 2020). Moreover, appearance‐focused and body‐acceptance messages often coexist within the same ‘body‐positive’ posts, creating tensions that may paradoxically harm body image (Trekels 2025). Due to emphasising the importance of appearance and attractiveness, BoPo has been criticised for drawing women's attention to their bodies and increasing their self‐objectification (Cohen, Fardouly, et al. 2019). Self‐objectification may be particularly triggered by visual depictions of female bodies (Cohen, Fardouly, et al. 2019; Taylor et al. 2023), as even non‐idealised diverse portrayals can heighten women's focus on appearance (concerns), despite their body‐positive framing (Betz and Ramsey 2017).

Building on the Objectification Theory (Fredrickson and Roberts 1997), this study explored whether BoPo posts on Instagram that portray female bodies and body‐positive text—compared to those that convey only body‐positive text—reinforce state self‐objectification for young adult women. Women are more often objectified in our society, being portrayed primarily as ‘bodies’, with their appearance framed as the central and defining aspect of their identity (Fredrickson and Roberts 1997). This translates to BoPo being predominantly posted by women and aimed at women (Rodgers et al. 2022). Clarifying whether BoPo posts displaying female bodies and text increase women's self‐objectification compared to textual‐only BoPo could help fulfil the original purpose of BoPo, which is to help followers accept and be satisfied with their bodies. These findings could also be practically applied to prevent negative body image by guiding users on social media to avoid certain types of BoPo while engaging with others.

### 1.1 BoPo and Self‐Objectification

Recently, social media platforms have seen the emergence of BoPo content. Some research suggests that most BoPo is consumed on Instagram (Stevens and Griffiths 2020), encompassing different types of content, such as ‘Instagram vs. reality’ posts, sexualised and non‐sexualised content, posts emphasising positive emotions and posts featuring only text without depicting bodies (Rodgers et al. 2023, 2024). While BoPo can assist followers in achieving a better body image and mitigating the influence of conventional beauty ideals (Cohen, Fardouly, et al. 2019; Rodgers et al. 2022), BoPo has been criticised for continuing to heavily emphasise appearance and consequently, reinforce self‐objectification (Cohen et al. 2021).

The effects of BoPo on self‐objectification can be explained by the Objectification Theory (Fredrickson and Roberts 1997), which posits that women are often viewed as bodies that are evaluated and exist for the pleasure of others. Consequently, women may experience heightened self‐objectification, characterised by the adoption of the view of oneself as an object to be evaluated, internalising the third‐person perspective (Fredrickson and Roberts 1997), and appreciating oneself for appearance rather than competencies (Daniels et al. 2020). This theory implies that, by commenting on and depicting female bodies, the media highlight the importance of appearance, which leads to the objectifying view of women and increases their self‐objectification. The Objectification Theory (Fredrickson and Roberts 1997) has been applied to new media (Cohen, Fardouly, et al. 2019), where the use of visually‐oriented social media platforms (e.g., Instagram) and participation in appearance‐related activities, such as posting selfies, viewing others' photos and reading comments about appearance, have been associated with increased self‐objectification (Wang et al. 2021; Fardouly et al. 2018).

Like other appearance‐focused social media content, BoPo incorporates objectifying elements, potentially increasing women's self‐objectification (Cohen, Fardouly, et al. 2019). However, the specific features of BoPo posts that may reinforce self‐objectification remain largely underexplored, yet understanding them is crucial for protecting women's body image. One key factor appears to be the presence of body depictions within posts (Cohen et al. 2021). Notably, approximately two‐thirds of BoPo content features women's bodies or at least some parts of them (Cohen, Irwin, et al. 2019). Compared to purely textual BoPo, posts depicting female bodies can be considered more objectifying, as they position women's bodies to be looked at and evaluated. This kind of presentation of bodies has been known to draw attention to appearance and elicit self‐objectification in the media context (Calogero 2012).

The existing research on this issue within BoPo remains scarce and inconclusive, some finding increased self‐objectification (Betz and Ramsey 2017; Vendemia et al. 2021) and negative body image (Rodgers et al. 2024; Rutter et al. 2024), while others have found no such effects (Cowles et al. 2023). Nevertheless, viewing appearance‐focused content online in general (Karsay et al. 2018) and on social media (Wang et al. 2025)—platforms well‐known for proliferating body imagery—has been linked to heightened self‐objectification. While numerous experimental studies have shown that exposure to idealised body depictions increases self‐objectification (Gurtala and Fardouly 2023; Prichard et al. 2023), even realistic portrayals of larger bodies can elevate it (Betz and Ramsey 2017; Fardouly et al. 2024). Viewing such images may redirect women's attention to their bodies (Anschutz et al. 2009), heighten body‐related anxiety (Swami and Smith 2012), and consequently reinforce self‐objectification.

### 1.2 Current Study

As existing research hints that exposure to BoPo content elicits self‐objectification among women (Cohen, Fardouly, et al. 2019; Vendemia et al. 2021), portrayals of bodies within these posts have been suggested as a potential driving factor (Cohen et al. 2021). This question has scarcely been investigated so far. Drawing upon the Objectification Theory (Fredrickson and Roberts 1997), the present study examined whether women exposed to BoPo with text accompanied by imagery of female bodies experience a higher level of state self‐objectification than women exposed to BoPo with text content alone. We also controlled for: trait self‐objectification, as women with high trait self‐objectification may be more sensitive to objectification cues and subsequently elevated state self‐objectification (e.g., Tiggemann and Boundy 2008; Vandenbosch et al. 2015); negative mood, which can lead to thinking about the size of our bodies or negative feelings about the body (Haedt‐Matt et al. 2012), and potentially increase state self‐objectification; and attitudes towards BoPo (Cohen et al. 2021), which may determine the reactions to viewed BoPo, as well as state self‐objectification. We hypothesised:

Hypothesis 1 *Women who view BoPo with text accompanied by female imagery have higher self‐objectification than women who view BoPo with text content alone*.

## 2 Method

### 2.1 Participants

The participants were 158 women aged 18–29 (M = 21.6, SD = 2.4). The majority were students (89%, of which only 14% were psychology students). Only 10% were employed; 1% did not indicate their employment status. Most participants (94%) were Czech, 4% were Slovak, and the rest were of a different or unspecified nationality. Of the initial 266 responses, 108 participants were excluded: 63 due to not completing any measurement, 29 due to not completing the Ten Statements Test (TST), 15 because they did not meet the participation criteria (age, gender), and one participant because of the retrospective withdrawal of consent to participate. The data are available at https://osf.io/ewkj8/overview.

### 2.2 Materials

The experimental groups differed in the type of BoPo posts they viewed. Participants saw either 10 BoPo Instagram posts containing body‐positive text and photos of female bodies (‘BoPo with text accompanied by imagery of female bodies’) or 10 BoPo Instagram posts containing only text (‘BoPo with text content alone’). The images depicted women whose bodies were (partially) exposed (e.g., in underwear or tight clothing). Most of the images also included the women's faces. Both groups viewed the same posts; the only difference was the presence of female body images (see Appendix E).

We developed the stimuli based on authentic posts from five authors of BoPo on Instagram. As the posts included images of the authors' bodies, we requested their permission to use their posts. Only one author provided consent. Consequently, we edited the posts from the other authors to display women from freely available licensed sources. We selected photos that closely matched the original author's body shape and pose to show the same body parts. Given that some respondents might recognise these accounts and realise that the original author's appearance was different, we also changed the account name and profile photo for all the posts (except those from the consenting author, Danielle Self Love Fitness Coach n.d.). For posts in Czech, we created an account named ‘mej.se.rada’ (which can be translated as ‘love.yourself’). For the English posts, we unified them under a fictional account named ‘body.positivity.movement’.

### 2.3 Procedures

The study had a between‐subject experimental design with two groups—participants saw either BoPo with text accompanied by the imagery of female bodies, or BoPo with text content alone. Because the study originated from a diploma thesis, a formal approval by a research ethics committee was not required under Czech regulations. Prior to data collection, the ethical aspects of the study were reviewed using the departmental checklist for diploma theses involving empirical research (Appendix A), and all aspects were assessed as non‐problematic. The study was preregistered: https://osf.io/am2v8/overview.

Conducting a power analysis in G*Power, we estimated a required sample size of 142 participants for multiple linear regression with four predictors (i.e., an experimental condition as a categorical predictor and the three controls). Data collection took place via Facebook and Instagram. The questionnaire was shared through the profile of the first author, several profiles of students assisting with the research, and the profile of a psychologist influencer. In addition, it was posted in various social media groups (e.g., a group for first‐year university students). There were no incentives offered to participants.

Respondents were first introduced to the research aim and participation conditions (as described in Appendix B). After giving their consent to participate, participants answered the background questions and evaluated their current mood and trait self‐objectification. They then received instructions to view 10 Instagram posts carefully. The duration of the exposure to BoPo posts was not restricted. Immediately after viewing the posts, participants completed questionnaires to measure state self‐objectification and attitudes towards BoPo. The final section was a debriefing, where participants learned the study's purpose. At the start, the questionnaire was ended for 15 participants who were either of other genders than women or out of the desired age range. Thirty‐three women were not included in the final sample as they did not fill out the Ten Statement Test, a measure of state self‐objectification. Additionally, one participant was not included because she withdrew her consent to participate after reading the debriefing (the full version is available in Appendix H) at the end of the study.

### 2.4 Pilot Study

The pilot study consisted of interviews with five women aged 21–23. The aim of the pilot study was to choose the final sets of 10 BoPo posts for each condition and to check the understandability of posts and whether participants considered them to be BoPo. All participants were provided with a definition of BoPo (Cohen, Fardouly, et al. 2019, 1550), which they had available throughout the interview. Participants saw 60 BoPo posts (30 for each experimental condition). Participants were then asked whether each post was BoPo and to assess its clarity, comprehensibility, and understandability (especially English posts). For posts containing images of bodies, participants were asked if the author and her appearance disrupted the post's message and whether it still qualified as a BoPo post. Subsequently, we selected 10 final posts that the participants included among their top 10 choices and agreed that they promoted body positivity. Additionally, we took into account participants' comments, such as whether they perceived a post as promoting obesity or as being too long text‐wise. Since such perceptions could interfere with the studied effect on state self‐objectification, these comments were among the decisive factors determining which posts were included in the final selection.

### 2.5 Measures

#### 2.5.1 Negative Mood

We used a 100‐point horizontal visual analogue scale to measure negative mood (Tiggemann and Andrew 2012). The question ‘How do you feel right now?’ was asked with five options: happy, anxious, confident, angry, and depressed. Each emotion was scored on a scale from ‘none’ to ‘very much’. Responses for ‘happy’ and ‘confident’ were reverse‐coded. The overall score was equal to the mean of the scores. The scale demonstrated fairly good internal consistency (ω = 0.85, 95% CI = 0.81; 0.88). Yet, the single‐factor confirmatory factor analysis pointed to some results outside of the standardly suggested criteria (SRMR = 0.05, *χ* ^2^(5) = 17.19, *p* = 0.004, RMSEA = 0.15, 90% CI = 0.08; 0.23, TLI = 0.88). The full wording of the scales and its Czech translation is provided in Appendix C.

#### 2.5.2 Trait Self‐Objectification

We used the Likert version of the Self‐Objectification Questionnaire (*LSOQ*; Wollast et al. 2021, full wording and its Czech translation provided in Appendix D), which has 10 items—five focused on appearance (i.e., weight, sex appeal, physical attractiveness, having firm/sculpted muscles, measurements) and five focused on competence (i.e., health, strength, energy level, physical fitness, physical coordination). Respondents were asked to rate the influence of each attribute on their body image with an 11‐point Likert scale (poles: ‘*low impact*’, ‘*high impact*’). The final trait self‐objectification score was calculated by subtracting the average rating of the competence‐related items from the average rating of the appearance‐related items. Higher scores indicated higher levels of trait self‐objectification. The scale showed satisfactory internal consistency (ω = 0.78, 95% CI = 0.74; 0.83). Based on the study by Wollast et al. (2021), we assumed a two‐factor model with the appearance and competence factors. However, the results were rather unsatisfactory (SRMR = 0.10, *χ* ^2^(35) = 207.16, *p* < 0.001, RMSEA = 0.14, 90% CI = 0.12; 0.16, TLI = 0.72). Considering that the original scale by Fredrickson et al. (1998) should be unifactorial, we tested this structure too, but it performed worse (SRMR = 0.10, *χ* ^2^(35) = 207.16, *p* < 0.001, RMSEA = 0.16, 90% CI = 0.14; 0.18, TLI = 0.62). Since both trait self‐objectification and negative mood were covariates and not the primary outcome variables, we included them in the analysis. Still, we conducted an additional robustness check to address any influence on the results.

#### 2.5.3 State Self‐Objectification

##### 2.5.3.1 Ten Statement Test (TST)

We measured state self‐objectification with an adapted 10‐item version of the Twenty Statement Test (Fredrickson et al. 1998). Participants were asked to describe themselves using 10 statements that begin with ‘I am’. Their responses were coded into six categories: body shape and size; other physical appearance; physical competence; traits or abilities; states and emotions; mixed or uncodable. The two coders reached satisfactory inter‐rater agreement (Cohen's *κ* = 0.78, 95% CI = 0.71; 0.86). The final score corresponded to the number of responses that fell into the first two categories, which represent self‐objectification. Scores ranged from 0 to 10. Higher scores indicated higher state self‐objectification. The average score was quite low, and almost half of the participants had a score of zero.

##### 2.5.3.2 Body Surveillance Subscale

Due to the possibility that participants would not complete the TST, especially in the online survey design, we also measured state self‐objectification with the Body Surveillance subscale of the Objectified Body Consciousness Scale (OBCS; McKinley and Hyde 1996). While this scale typically measures trait self‐objectification, we added ‘right now’ to each statement to assess state self‐objectification, consistent with prior research (e.g., Breines et al. 2008; Tiggemann and Andrew 2012). Eight statements were rated on a 7‐point Likert scale (1 = *strongly disagree*, 7 = *strongly agree*). The state self‐objectification score corresponded to the sum of the scores across all statements, ranging from 8 to 56. Higher scores represented higher levels of state self‐objectification. The internal consistency of the scale was satisfactory (ω = 0.83, 95% CI = 0.80; 0.87). The results of the confirmatory factor analysis were generally satisfactory (SRMR = 0.06, *χ* ^2^(20) = 63.40, *p* < 0.001, RMSEA = 0.11, 90% CI = 0.08; 0.14, TLI = 0.88), except for slightly less‐than‐ideal *RMSEA* and *TLI*. The full wording of both self‐objectification scales and their Czech translations are provided in Appendix F.

#### 2.5.4 Attitude Towards BoPo

Given the lack of an available scale for attitude towards BoPo, we developed our own, consisting of three statements based on the cognitive, affective, and behavioural attitudinal components (Eagly and Chaiken 1993). All items were formulated to reflect a positive attitude towards BoPo, which could be observed in three areas: positive thoughts about BoPo, positive emotions evoked by BoPo, and actions that reflect positive attitudes, such as an active choice of BoPo that signals support, engagement, or endorsement of BoPo. The statements (i.e., ‘Looking at body positivity posts can lead to a more positive perception of my body’; ‘Body positivity posts evoke positive emotions in me’; ‘I follow body positivity accounts on some social media platforms’; full wording and translation are available in Appendix G) were rated on a 5‐point Likert scale (1 = *definitely disagree*, 5 = *definitely agree*). The resulting score was equal to the mean of the responses to the items. The scale demonstrated satisfactory reliability (ω = 0.77, 95% CI = 0.71; 0.83) and just‐identified factor model (SRMR = 0.00, *χ* ^2^(0) = 0.00, RMSEA = 0.00, 95% CI = 0.00; 0.00, TLI = 1.00).

### 2.6 Data Analysis

The ordinal regression model was used instead of planned linear regression because the assumption of continuous data was violated: more than half of the participants scored zero on the state self‐objectification variable, measured by the TST, and the maximum was five (instead of the theoretical maximum of 10). Beyond preregistration, we additionally examined the effect of the female body in BoPo on state self‐objectification as measured by the OBCS due to TST's floor effect to cross‐validate our results. This was done with a sample consisting of 187 women (Subsample 2; M = 21.6, SD = 2.3), drawn from the total sample collected for this study. The original sample collected for this study consisted of 191 women aged 18–29 (M = 21.6, SD = 2.3), from which 158 women aged 18–29 (Subsample 1; M = 21.6, SD = 2.4) were included in the main preregistered analysis with TST, as 33 women did not meet the inclusion criteria of completing the TST items. Given that these criteria were not relevant for the additional analysis with the OBCS, the data from these previously excluded women were included in this analysis, except for four women who did not fill out the OBCS items. Thus, compared to the main sample described in the Participants section, the sample for this analysis included an additional 29 women who were excluded from the original sample due to not completing the TST but completed the OBCS items.

Furthermore, we explored whether trait self‐objectification, pre‐exposure negative mood, and attitudes towards BoPo moderated the impact of female body depictions within BoPo posts on state self‐objectification, as measured by the OBCS subscale. The moderation term (created by multiplying the predictor and the moderator) was entered into the regression analysis. For significant moderations, the effect of BoPo on state self‐objectification was then estimated for three groups based on the moderator's level: −1 SD, mean, and +1 SD.

We reported the results from the three models. The first and the second were used to test the preregistered hypothesis and the role of control variables (i.e., trait self‐objectification, negative mood, attitude towards BoPo). In the first model on Subsample 1, which was a hierarchical ordinal regression, we used the control variables, the experimental group variable, and the state self‐objectification measured by TST as a dependent variable. Due to the violation of the proportional odds assumption, we additionally conducted a series of three binomial logistic regressions. The second model on Subsample 2 was a hierarchical linear regression, which included the same predictor variables and the state self‐objectification measured by the OBCS subscale as a dependent variable. Using Subsample 2, the last hierarchical linear regression model included all previously mentioned predictor variables and the state self‐objectification measured by the OBCS subscale as an outcome and explored the moderation effects of the control variables. In all analyses, the only outcome variable was state self‐objectification (measured by TST or OBCS); trait self‐objectification, negative mood, and attitudes towards BoPo were used as predictors or moderators.

## 3 Results

### 3.1 Descriptive Statistics

Zero‐order correlations and descriptive statistics are presented in Table 1. State self‐objectification measured by the TST showed a significant floor effect and did not correlate with other variables. However, state self‐objectification measured by the OBCS correlated positively with trait self‐objectification and negative mood.

**TABLE 1:** Descriptive statistics and zero‐order correlations between the measured variables.

	M	SD	(1)	(2)	(3)	(4)	(5)
(1) State self‐objectification (TST)	0.9	1.1					
(2) State self‐objectification (OBCS)	31.1	9.7	0.14				
(3) Trait self‐objectification	−0.3	2	0.14	0.36*			
(4) Negative mood	37.2	20	0.08	0.34*	0.09		
(5) Attitude towards BoPo	3.4	0.9	−0.05	0.02	−0.01	0.01	

* < 0.05.

### 3.2 The Impact of the Female Bodies Within BoPo on State Self‐Objectification

First, we tested our hypothesis using self‐objectification as measured by TST (as preregistered, using Subsample 1) in an ordinal regression model. The results, summarised in Table 2, showed that the experimental condition did not significantly predict state self‐objectification. Thus, the hypothesis that depicting female bodies within BoPo leads to higher state self‐objectification was not supported. Similarly, none of the covariates significantly predicted state self‐objectification.

**TABLE 2:** Ordinal regression, dependent variable: Self‐objectification measured by TST.

Predictor	B	OR	95% CI OR	*p*	*R* ^2^
Experimental condition	−0.04	0.96	0.53; 1.72	0.951	0.00
Trait SO	0.11	1.12	0.96; 1.30	0.159	0.00
Negative mood	−0.00	1.00	0.98; 1.02	0.983	0.00
Attitude	0.04	1.04	0.76; 1.43	0.801	0.01

Due to the violation of the proportional odds assumption, we additionally conducted a series of three binomial logistic regressions, where respondents with state self‐objectification values of 0 and 1 (i.e., negligible self‐objectification); 2 and 3 (i.e., some self‐objectification); and 4 and 5 (i.e., substantial self‐objectification) entered the regression. However, only six respondents fell into the last group, and only one had a value of 5 (which violates the proportional odds assumption), so evaluating the regression coefficients was meaningful only for the first two models. In both models, the experimental condition still emerged as a non‐significant predictor of state self‐objectification (model with negligible state self‐objectification: B = 0.33; *p* = 0.407, OR = 1.38, 95% CI OR = 0.64; 2.92, *R* ^2^ = 0.05; model with some state self‐objectification: B = 0.02; *p* = 0.979, OR = 1.02, 95% CI OR = 0.22; 4.72, *R* ^2^ = 0.01).

We also tested the effect of depicting the female body within BoPo using the OBCS to measure state self‐objectification (the results are summarised in Table 3), using Subsample 2. Again, the experimental condition was not a significant predictor of state self‐objectification. Trait self‐objectification and negative mood were significantly and positively related to state self‐objectification. The attitude towards BoPo did not significantly predict state self‐objectification.

**TABLE 3:** Linear regression, dependent variable: Self‐objectification measured by OBCS.

Predictor	B	SE	*β*	95% CI *β*	*p*	*R* ^2^	Δ*R* ^2^
Experimental condition	1.69	1.41	0.17	−0.11; 0.46	0.233	0.01	0.01
Trait SO	1.78	0.33	0.37	0.23; 0.50	< 0.001	0.14	0.14
Negative mood	0.15	0.03	0.30	0.17; 0.43	< 0.001	0.23	0.09
Attitude	0.20	0.66	0.02	−0.11; 0.15	0.757	0.23	0.00

### 3.3 Moderating Role of Trait Self‐Objectification, Negative Mood, and Attitude Towards BoPo

Furthermore, we explored whether trait self‐objectification, pre‐exposure negative mood, and attitude towards BoPo moderated the impact of the depictions of the female body within BoPo posts on state self‐objectification as measured by the OBCS subscale. Trait self‐objectification (B = −0.32, SE = 0.65, *β* = −0.07, *p* = 0.623), negative mood (B = −0.05, SE = 0.07, *β* = −0.11, *p* = 0.424), and attitude towards BoPo (B = −1.11, SE = 1.33, *β* = −0.11, *p* = 0.403) did not moderate the relationship between the experimental condition and state self‐objectification. Similar results appeared when using the preregistered scale of state self‐objectification (TST) for trait self‐objectification (B = −0.05, SE = 0.09, *p* = 0.618) and negative mood (B = −0.01, SE = 0.01, *p* = 0.243). Nonetheless, the moderation of attitude towards BoPo was statistically significant (B = −0.42, SE = 0.19, *p* = 0.031). However, its effect was not that pronounced and not significant at the individual levels of attitude (low (−1 SD): B = 0.31, SE = 0.25, *p* = 0.211; average: B = −0.05, SE = 0.18, *p* = 0.777; high (+1 SD): B = −0.42, SE = 0.25, *p* = 0.098). Yet, these results could suggest that the female body in BoPo increases state self‐objectification for women with a more negative attitude towards BoPo. Contrastingly, when the attitude is more positive, depictions of female bodies might lead to lower state self‐objectification.

### 3.4 Robustness Check Analysis

Because of poor fits for the trait self‐objectification and negative mood scales and the just‐identified model for attitude towards BoPo, we additionally ran the main models (i.e., linear regression with state self‐objectification measured by OBCS, ordinal regression with state self‐objectification measured by TST) without these variables to support the adequacy of our conclusions. These additional analyses supported our previous results. Exposure to female body portrayals did not lead to higher state self‐objectification in both models, OBCS (B = 1.69, *β* = 0.17, 95% CI *β* = −0.11; 0.46, *p* = 0.233) and TST (B = −0.02, OR = 0.98, 95% CI OR = 0.55; 1.75, *p* = 0.951).

## 4 Discussion

The current study found that exposure to BoPo with text accompanied by imagery of female bodies did not result in heightened self‐objectification among young women. These results contrast presumptions derived from the Objectification Theory (Fredrickson and Roberts 1997) and research suggesting that body portrayals reinforce self‐objectification and body image concerns (Cohen et al. 2021; Rodgers et al. 2024) but align with a few existing studies on this topic (Cowles et al. 2023; Taylor et al. 2023). Building on this line of research, the present study suggests that mere body portrayals may not be the primary triggers of self‐objectification in BoPo on Instagram.

A couple of possible explanations come forward. The effects on self‐objectification may depend on how the body is portrayed, as depictions of unposed bodies that deviate from conventional beauty standards used in this study are likely to be perceived as less objectifying than idealised portrayals. Supporting this, Vendemia et al. (2021) found that exposure to sexualised and digitally modified BoPo content led to greater idealisation of conventional beauty standards compared to a control condition. We advocate for further investigation into the various ways of depicting female bodies within BoPo (e.g., (un)posed, (un)sexualised, appearance‐focused versus functionality context) and their impact on self‐objectification. Another possible explanation for the null findings could lie in the objectification potential within the BoPo text. Captions in BoPo usually comment on bodies, and even though participants in the control group did not see a female body, they did read about it. By highlighting the body, captions may reinforce self‐objectification, which would explain why women might have experienced elevated self‐objectification in both conditions. Such text could function as priming, activating the mental representation of the body and resulting in self‐objectification, as demonstrated by previous literature (Roberts and Gettman 2004; Vandenbosch et al. 2015). However, it must be emphasised that, given the design of the present study, we cannot conclude whether state self‐objectification increased or remained the same in both conditions.

A last explanation lies in the subjective perceptions of objectification. The Objectification Theory (Fredrickson and Roberts 1997) proposes that self‐objectification arises when a woman perceives herself as objectified, not whether she is actually objectified. Analogically, how much the posts are perceived as objectifying seems to be an important consideration for future research. Indeed, research in this area has emphasised the importance of subjective appraisals of sexualisation in forming the effects on state self‐objectification (Vendemia et al. 2021). We may also question whether people are still sensitive to human bodies shown in social media, as previous studies suggested desensitisation to these posts due to the pervasiveness of bodies in their daily experience (e.g., Schreurs and Vandenbosch 2022). As a result, bodies in BoPo might not necessarily contribute to omnipresent self‐objectification.

While previous research has suggested that individuals higher on trait self‐objectification (Vandenbosch et al. 2015) and negative mood (Haedt‐Matt et al. 2012) tend to be more sensitive to the objectifying stimuli, the effects of female body depictions on state self‐objectification were not moderated by these variables. However, with the Ten Statement Test, attitude towards BoPo was found to be a significant moderator: exposure to BoPo featuring female bodies led to heightened self‐objectification in women holding a more negative attitude towards BoPo, whereas a more positive attitude led to less self‐objectification. Women with a positive attitude may focus on positive body messages (e.g., body care, body functionality) rather than negative messages, such as the importance of appearance. Indeed, women who learn to focus on the functionality of their bodies rather than their appearance exhibit lower self‐objectification and better body image (Alleva et al. 2015). On the contrary, women showing negative attitudes towards BoPo may avoid its healing messages and fixate more strongly on body depictions. Still, the moderating effect of attitude was evident only when using the TST and not with the OBCS measure of self‐objectification. Further investigation is needed into the role of attitudes towards BoPo and its effects on body image.

The present study addressed the growing calls to shed light on the characteristics of BoPo content that may heighten self‐objectification and contribute to negative body image (Cohen et al. 2021; Fardouly et al. 2018). Our results showed that portrayals of female bodies may not be the driving factor behind elevated self‐objectification. While distinguishing posts based on whether they increase self‐objectification appears valuable for creators and followers of BoPo, this study showed that BoPo posts portraying bodies should not be treated as reinforcing self‐objectification without further investigations and guided to be avoided. Follow‐up research is recommended to acknowledge and explore the objectification potential of body‐positive texts and subjective perceptions of objectifying information in the posts.

### 4.1 Limitations and Future Directions

One limitation of the experimental design is that the control group of text‐only BoPo did not include a neutral image to strictly isolate the effect of body imagery. Although the posts used in this study reflected authentic BoPo content from Instagram (Cohen, Irwin, et al. 2019), future research should consider including neutral images to control for the potential influence of mere exposure to any image. Another issue to consider is the ecological validity, as participants viewed the posts outside their real Instagram environment, where posts, comments, and likes come from friends or known influencers. While participants may have identified with the unknown authors, the posts might have been less powerful in influencing self‐objectification than those authentically encountered on social media platforms. Furthermore, self‐objectification induced by engagement with BoPo may unfold over a longer period than what can be captured in a brief experimental exposure. In this regard, employing an experimental ecological momentary assessment, which allows participants to be exposed to BoPo content in a more naturalistic setting and tracks self‐objectification development over time, seems to be a promising direction for future research. An additional limitation concerns the absence of attention checks in the questionnaire. Given the limited control over data collection conducted via social media platforms, it is possible that some participants were not fully attentive to the stimuli or questionnaire items, which may pose a potential threat to the overall validity.

Furthermore, one of the limitations of this study concerns the Ten Statements Test (TST). We uncovered three potential issues: unclear instructions, questionable validity, and coding challenges. First, the intentionally vague instructions to capture spontaneous self‐descriptions may cause confusion. For example, one participant responded ‘I am confused because I do not know what these sentences should say about me’. Such ambiguity may result in responses that do not accurately reflect participants' current self‐objectification. Second, the TST showed weak correlations with the OBCS measure of state self‐objectification and did not correlate with trait self‐objectification or negative mood, raising concerns about its validity. Third, many responses contained both objectifying and non‐objectifying elements, forcing us to categorise them as ‘mixed’ or ‘uncodable’. Unlike previous studies (e.g., Cohen, Fardouly, et al. 2019), which coded such responses as objectifying, our stricter approach may have underestimated self‐objectification levels, potentially explaining discrepancies between TST and OBCS results. Despite its frequent use to measure state self‐objectification, these issues highlight the need to reevaluate the TST's validity, as Cowles et al. (2023) reported a similar issue with this scale.

Another limitation concerns the measurement of attitude towards BoPo, because the scale consists of only three items and has not yet been formally validated. Although the items have originated from the classical tripartite model of attitudes encompassing affective, cognitive, and behavioural components (Eagly and Chaiken 1993), behaviours may not depend consistently on affects and cognitions across different contexts (Glasman and Albarracín 2006). Thus, the inclusion of a behavioural component may have partly captured users' exposure to body positivity content rather than solely their attitudinal stance. Such measurement may have artificially inflated the attitude scores for participants who follow body‐positivity accounts for different reasons (e.g., informational or social) rather than out of a positive attitude, and, conversely, deflated the scores for those who hold a positive attitude but do not follow such accounts, for instance, due to passive or minimal social media use. Also, alternative models of attitudes have been put forward recently that take into account the cost of different behaviours (Kaiser and Wilson 2019) or look at attitudes as a network of components rather than a common‐cause model (Dalege et al. 2016). Although attitudes towards BoPo were included as a covariate in the present study, future research that examines these attitudes more closely should consider alternative modelling approaches that may better capture their nature.

Lastly, we acknowledge that future research on BoPo effects should not be restricted to women. The impact of BoPo on the body image of men and other genders has not been studied extensively, though some BoPo are targeted at them. Apart from assuring gender diversity among the participants, future research should include BoPo posts that display different genders. Given that posts with images function through comparison and the internalisation of what is seen, the influence of BoPo may differ depending on the gender (in)congruency between the post and the viewer.

## 5 Conclusion

Although previous studies have reported increased self‐objectification following exposure to BoPo content (Cohen, Fardouly, et al. 2019), the present study suggests that the depiction of female bodies in such posts may not be the underlying trigger. The results also preliminarily indicated that women with negative attitudes towards BoPo may be more susceptible to heightened self‐objectification after viewing bodies featured in such posts. Consequently, follow‐up research should focus on how bodies are depicted in body‐positivity content and examine the objectification potential of body‐positive text to determine whether merely reading about appearance can increase self‐objectification. This study emphasises the need for further exploration of how specific elements of BoPo content contribute to self‐objectification, thereby deepening the understanding of its effects on body image in the social media context. In conclusion, the present study underlines that there is currently no need to encourage women who engage with the BoPo movement to avoid all posts depicting bodies, at least in terms of self‐objectification risk. Instead, prevention efforts and social media campaigns should focus on raising awareness about specific features within this content that may lead users to overemphasise appearance and feel negatively about their bodies despite the movement's positive intentions.

## Author Contributions

**Nina Krizova:** conceptualisation, data curation, formal analysis, investigation, methodology, writing – original draft. **Nikol Kvardova:** conceptualisation, investigation, methodology, project administration, supervision, writing – review and editing. **Hana Machackova:** funding acquisition, supervision, writing – review and editing.

## Funding

This work has been funded by a grant from the Programme Johannes Amos Comenius under the Ministry of Education, Youth and Sports of the Czech Republic from the project ‘Research of Excellence on Digital Technologies and Wellbeing CZ.02.01.01/00/22_008/0004583’ which is co‐financed by the European Union.

## Ethics Statement

The ethical aspects of the study were reviewed prior to data collection using the Masaryk University's ethical compliance checklist. As the research was primarily part of a diploma thesis, it was not submitted for additional approval by an ethics committee, as is permissible under Czech regulations. All participants provided written informed consent prior to participating. The study was performed in accordance with the ethical standards as set forth in the 1964 Declaration of Helsinki and its later amendments.

## Consent

Informed consent was obtained from all individual adult participants included in the study.

## Conflicts of Interest

The authors declare no conflicts of interest.

## Supporting information


**Appendix A.** Presents a checklist of ethical considerations reviewed before the study was conducted. The remaining Supporting Information provide the exact wording of the full survey as presented to participants, in the original order. Appendix B contains the informed consent form. Appendices C and D present the measures of negative mood and trait self‐objectification, respectively, in both English and Czech versions. Appendix E includes all body positivity stimuli used in the study. Appendices F and G present the measures of state self‐objectification and attitudes towards body positivity (in both Czech and English). Appendix H contains the debriefing administered at the end of the survey.

## Data Availability

The authors confirm that they have access to the data used in this study. The data are publicly available at: https://osf.io/ewkj8/overview.
