# Supplementary material for: Empowerment or Pressure? Exploring the Impact of Female Body Depictions in Body Positivity Instagram Posts on Self‐Objectification
Source: Int J Psychol. 2026 Apr 15;61(3):e70208. doi: 10.1002/ijop.70208 (PMC13083542; doi:10.1002/ijop.70208)
Supplement: Supplementary file 1 — Appendix A. Presents a checklist of ethical considerations reviewed before the study was conducted. The remaining Supporting Information provide the exact wording of the full survey as presented to participants, in the original order. Appendix B contains the informed consent form. Appendices C and D present the measures of negative mood and trait self‐objectification, respectively, in both English and Czech versions. Appendix E includes all body positivity stimuli used in the study. Appendices F and G present the measures of state self‐objectification and attitudes towards body positivity (in both Czech and English). Appendix H contains the debriefing administered at the end of the survey. [file IJOP-61-e70208-s001.docx]

**Supplementary Appendices**

**Supplementary Appendix A. Checklist for the Ethical Aspects of a Research Thesis for the Department of Psychology of (Anonymized) University**

The following checklist is intended as an approximate assessment of whether there are aspects relevant to research ethics in your final thesis. For each item, indicate whether it applies to the research conducted in your bachelor's/master's thesis (i.e., Yes or No). For each item that applies to your research:

(a) Describe in detail what it entails, and

(b) Explain how you have addressed this ethical aspect in your research.

If the answer to one or more items is YES, it is necessary to consult with your thesis supervisor about these items (including their detailed description and your proposal for addressing them). In more complex cases, your supervisor may recommend further consultation with individuals who represent the faculty in the Ethics Committee for Research at MU.

Completing the checklist and any subsequent consultations are necessary before commencing the research and before you begin assembling the research sample and collecting data.

1. Will I be working with personal data at any stage of the research (i.e., data that potentially allow for the identification of specific individuals)? This may include names, email addresses, phone numbers, postal addresses, and other contact information, like birthdate data. This category also includes photographs, video, and audio recordings of interviews (even if the name is not explicitly mentioned, a person may be identifiable based on appearance, voice, or certain facts mentioned in the interview). Similarly, materials obtained from social networks may fall into this category.

2. Will I approach individuals for participation in the research who are under 18 years of age or lacking legal capacity, or can it be assumed that they have a reduced ability to provide informed consent for their participation?

3. Will I approach vulnerable individuals for participation (e.g., those facing discrimination or socio-economic disadvantage, individuals in extreme life situations, individuals with serious physical or mental illness, victims of violence)?

4. Will I approach individuals who are in a dependent relationship with me in some form (e.g., my clients, subordinates, wards, students)?

5. Does the nature of the research preclude informing the participants in advance about the purpose and course of the research or obtaining their explicit consent to participate?

6. Will there be deception of the participants regarding the true purpose of the research at some stage, or will I interpret the results in a way that could potentially affect the participants, as it may be contrary to their expectations (e.g., presenting a research interview as a neutral examination of their beliefs but interpreting the results using potentially offensive terms such as prejudice, psychopathy)?

7. Will I use biomedical methods or methods on the boundary between behavioural and biomedical research (e.g., measuring heart rate or skin resistance, taking saliva samples, administering any substances)?

8. Will participation in the research impose a time burden on the participants (i.e., approximately >60 minutes), the need to travel for an extended period, the expenditure of their own financial or other resources, or other potential hardships?

9. Will participants be exposed to situations and activities that may not be pleasant for them (including performing boring tasks, deliberately inducing negative emotions)?

10. Will I inquire about sensitive topics from participants (e.g., in the areas of mental and health problems, sexuality, risky behaviour, or illegal behavior)?

11. Will I require the participants to engage in physical activity as part of the research?

12. Is there a risk (even a very small one) that participation in the research may harm participants (i.e., psychological, physical, material, or financial harm, damage their reputation or standing, harm personal relationships, employment, studies)?

13. Does the research include other ethically relevant aspects that do not fall under any of the previous points?

**Supplementary Appendix B. Informed consent**

My name is (Anonymized), and I am a psychology student at (Anonymized) University. I would like to request your participation in a research study that is part of my bachelor's thesis. The aim of my research is to investigate how so-called "body positivity" posts (content that seeks to counter the influence of unattainable beauty ideals and promotes the appreciation of all bodies) on Instagram influence body image.

Women aged 18 to 29 (inclusive) are eligible to participate in the study. In the research, you will be asked to complete questionnaires related to demographic information, your self-perception, and your current mood. First, I will ask you to fill out demographic information and two short scales. Subsequently, I will ask you to view 10 body positivity posts. Following that, the final part of the study will involve a short questionnaire to assess the attributes that are currently important to you, and your agreement with three statements. Overall, your participation in the research will take no more than 15 minutes.

The research is anonymous, and I will not collect any of your personal information. Participation in the research is voluntary and it is not associated with any rewards or risks.

Your participation is entirely voluntary. The questions are not mandatory, so you are not obligated to answer them. At any point during or immediately after the procedure, you have the right to withdraw your participation without providing a reason. In such a case, all of your data will be removed. Later withdrawal from the study is not possible because the collected data will be anonymous (i.e., it will not be possible to identify which data comes from you).

If you have any questions, you can contact me via email at: (Anonymized e-mail address)

Please confirm your consent to participate in the research by clicking the button below.

**Supplementary Appendix C. Measurement of negative mood**

*Original version*

happy, anxious, confident, angry, depressed

poles: not at all; very much

*Translation*

šťastně, úzkostně, sebejistě, naštvaně, depresivně

poles: vůbec ne, velmi

*Task*

Jak se právě teď cítíte?

0 = vůbec ne

100 = velmi

**Supplementary Appendix D. Measurement of trait self-objectification**

*Original version*

The questions below identify 10 different body attributes. We would like you to indicate the extent to which each of these body attributes has an impact on your physical self-concept. For each attribute, please indicate whether the attribute has an extremely low or extremely high impact on your physical self-concept.

From 1 (Low impact) to 11 (High impact)

1. Health
2. Weight
3. Strength
4. Sex appeal
5. Physical attractiveness
6. Energy level (e.g., stamina)
7. Having firm/sculpted muscles
8. Physical fitness
9. Measurements (e.g., chest, waist, hips)
10. Physical coordination

*Translation*

Níže je uvedených 10 různých tělesných vlastností. Uveďte, prosím, do jaké míry má každý z těchto tělesných atributů dopad na vaše tělesné sebepojetí (představa o vlastním těle).^[[1]](#footnote-1)^ U každého atributu uveďte, zda má extrémně nízký nebo extrémně vysoký dopad na vaše tělesné sebepojetí. Odpovídejte prosím v rozmezí od 1 (nízký dopad) do 11 (vysoký dopad).

1. zdraví
2. váha
3. síla
4. sex appeal
5. fyzická atraktivita
6. míra energie (např. výdrž)
7. silné/vypracované svaly
8. fyzická zdatnost
9. míry (např. hrudníku, pasu, boků)
10. fyzická koordinace

**Supplementary Appendix E. Body positivity content – experimental conditions**

| Posts not depicting female body | Posts depicting female body |
| --- | --- |
| 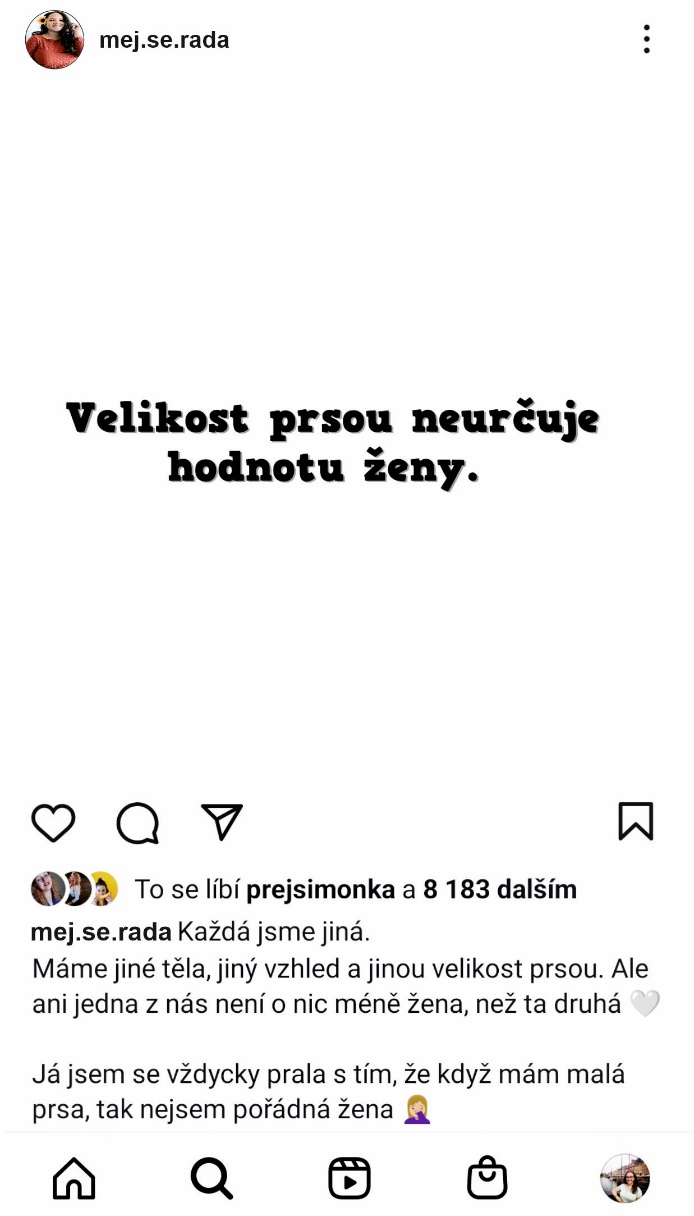 | 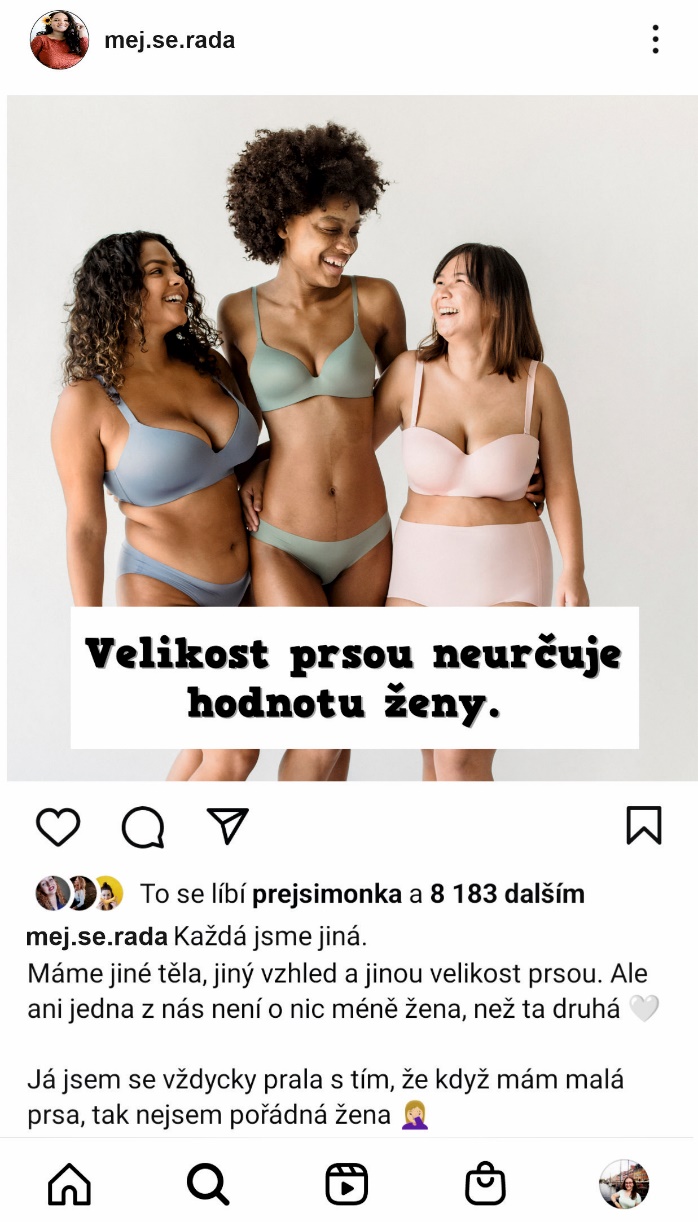 |
| 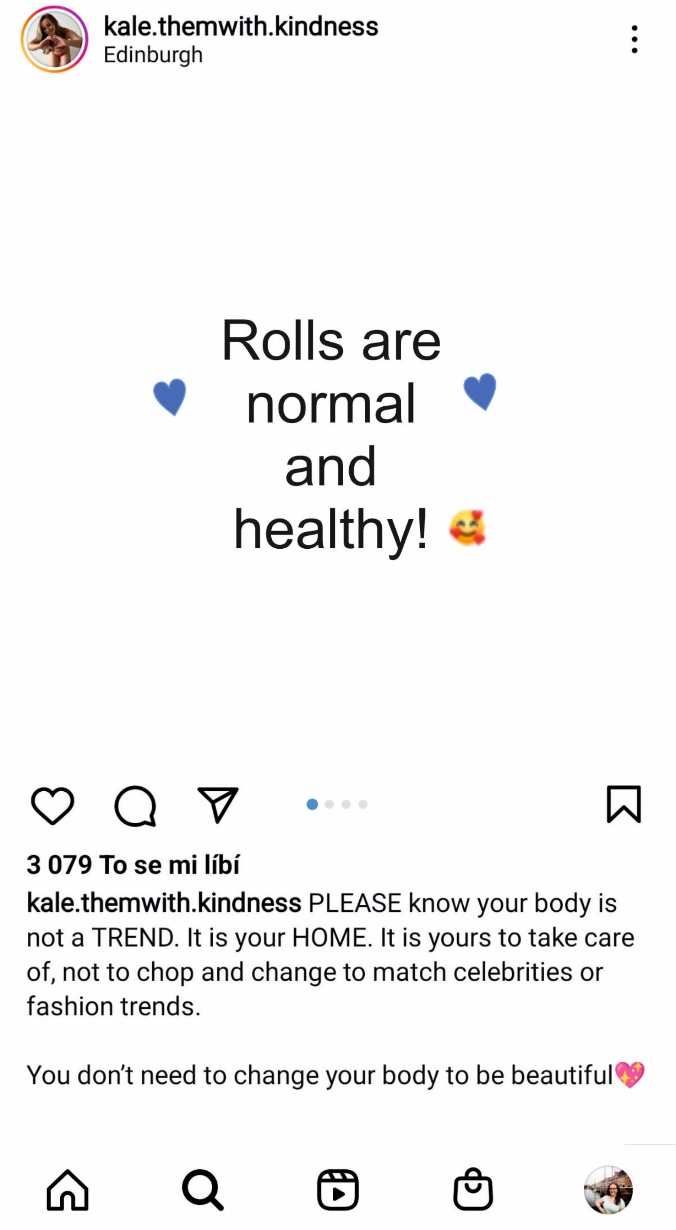 | 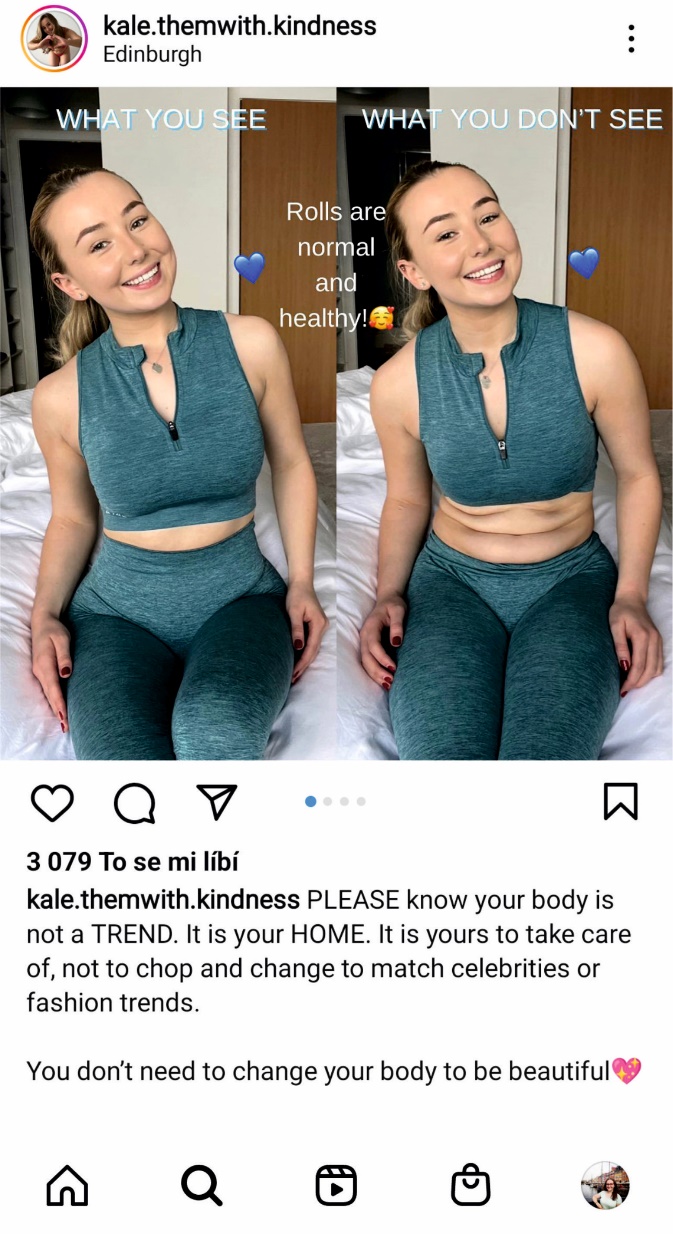 |
| 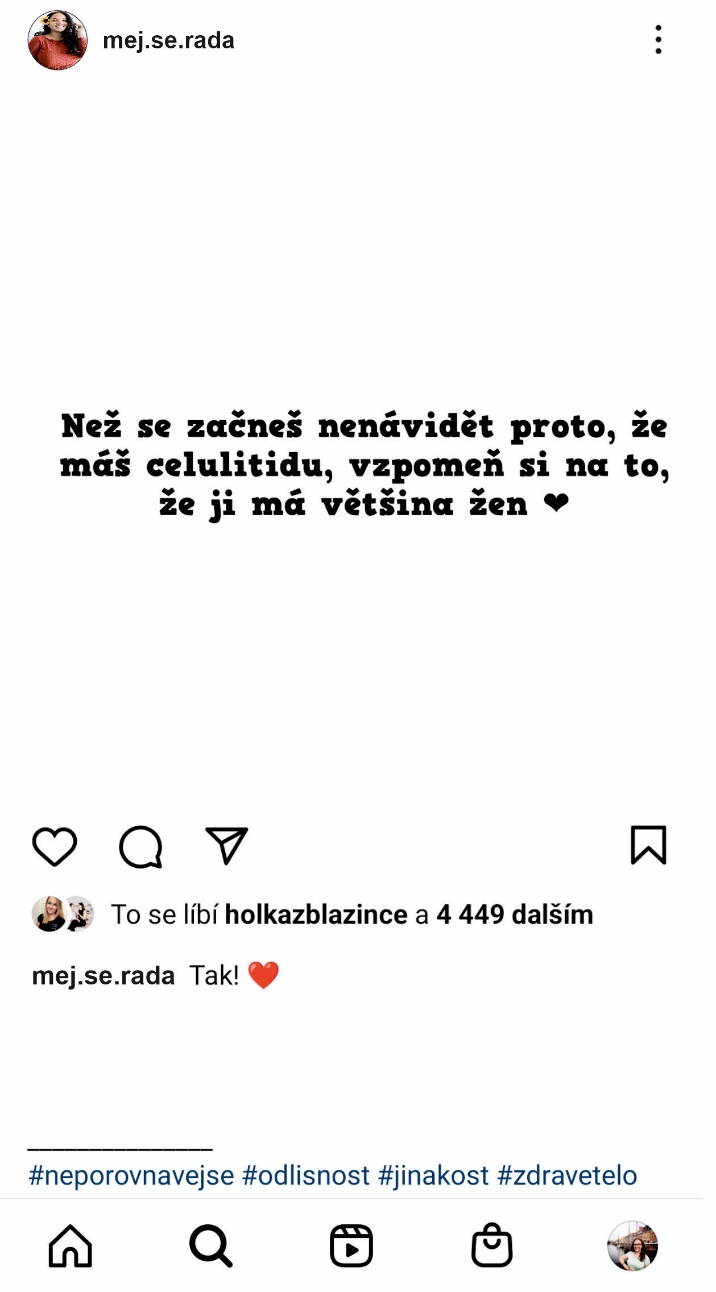 | 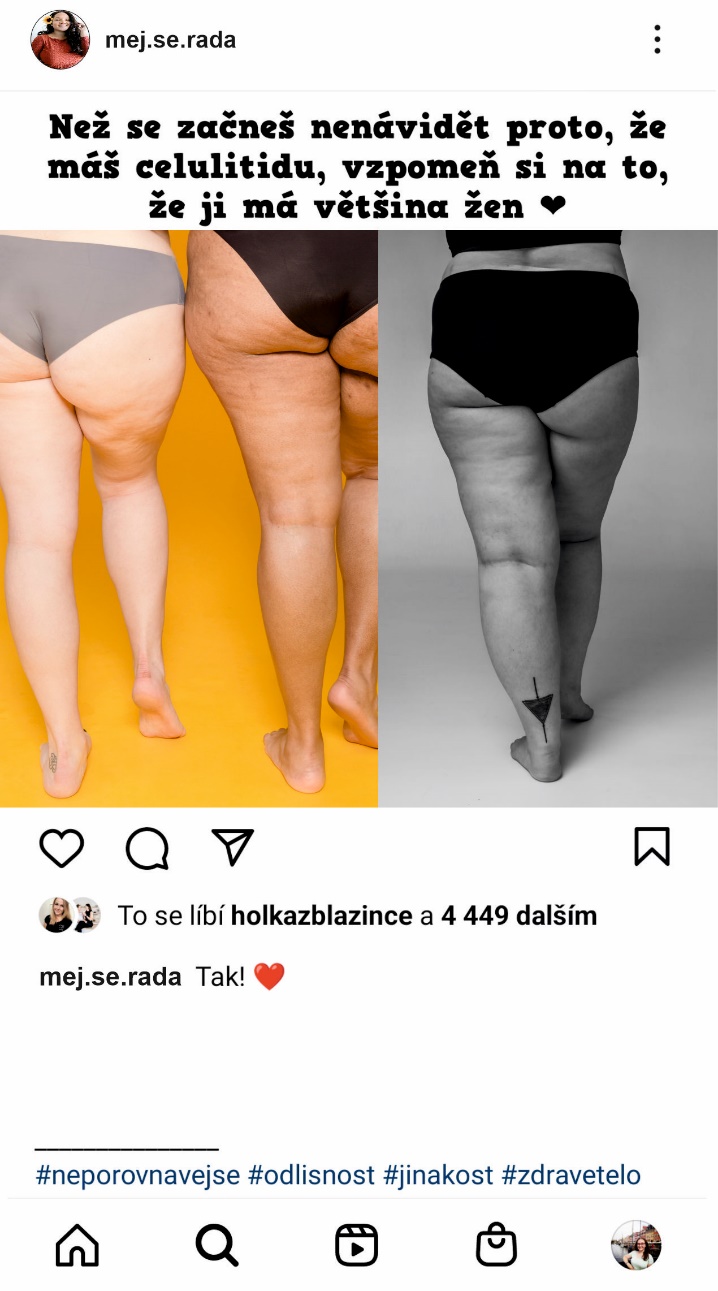 |
| 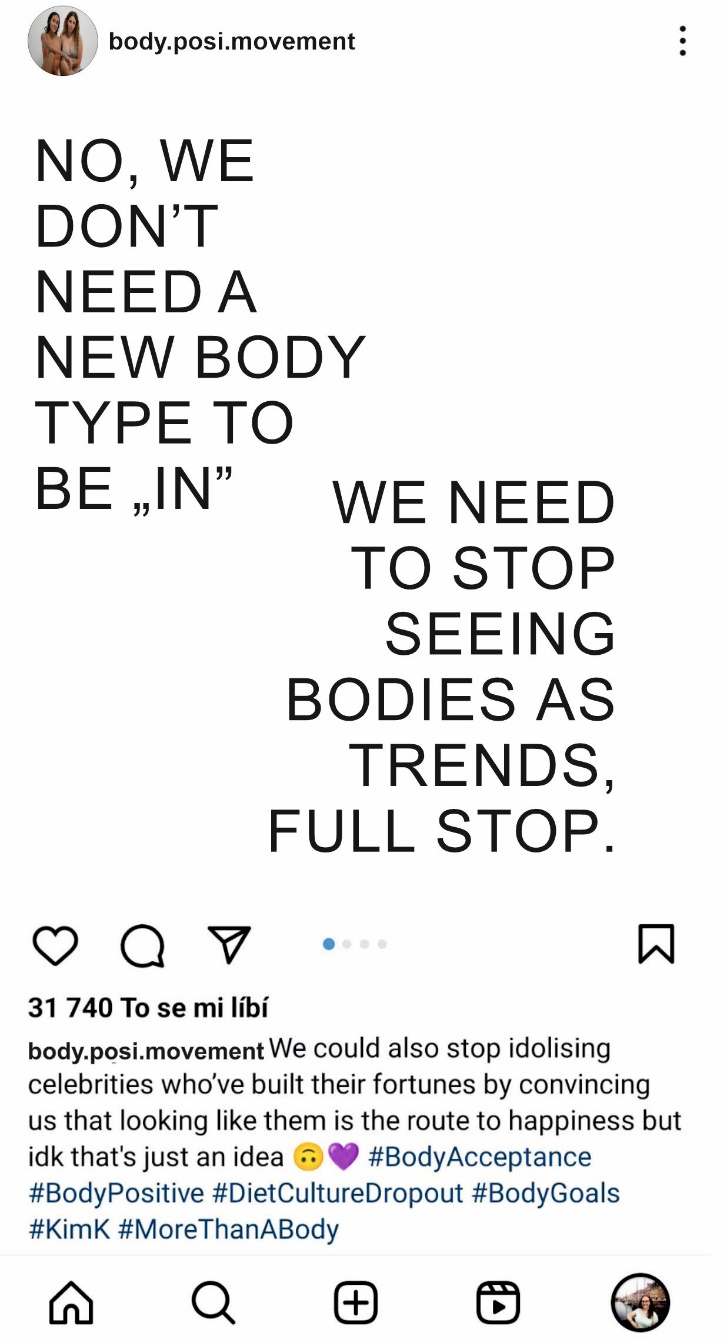 | 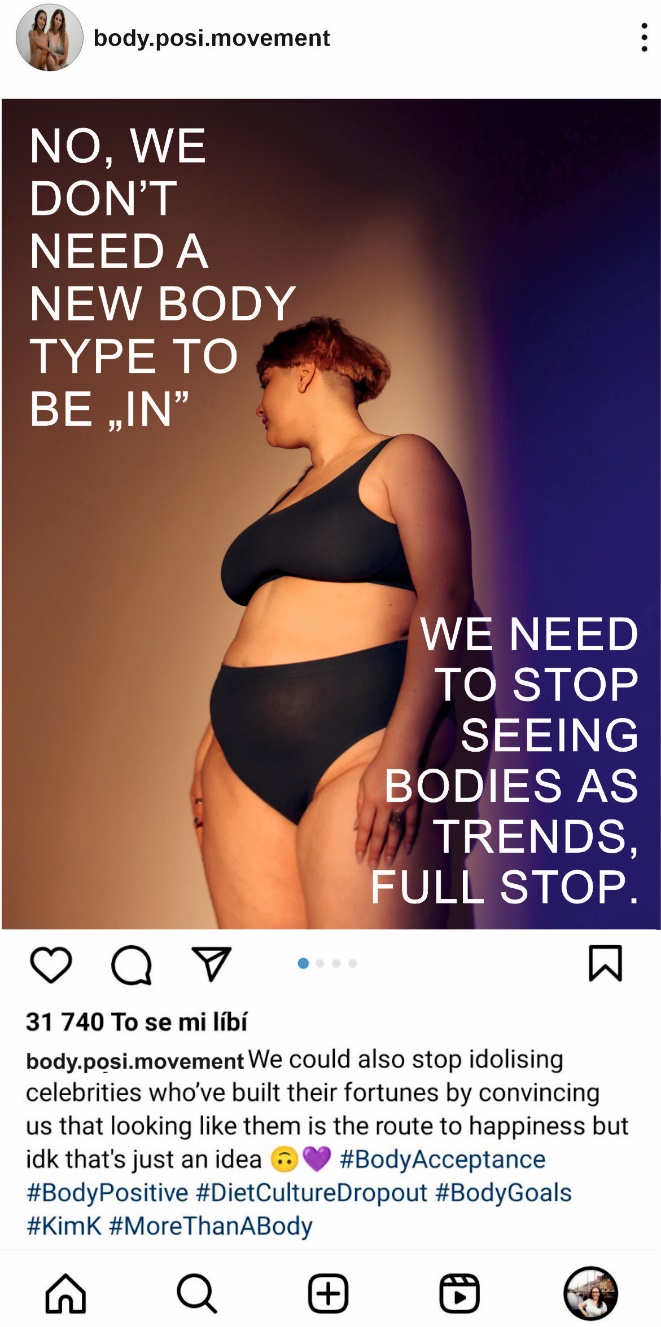 |
| 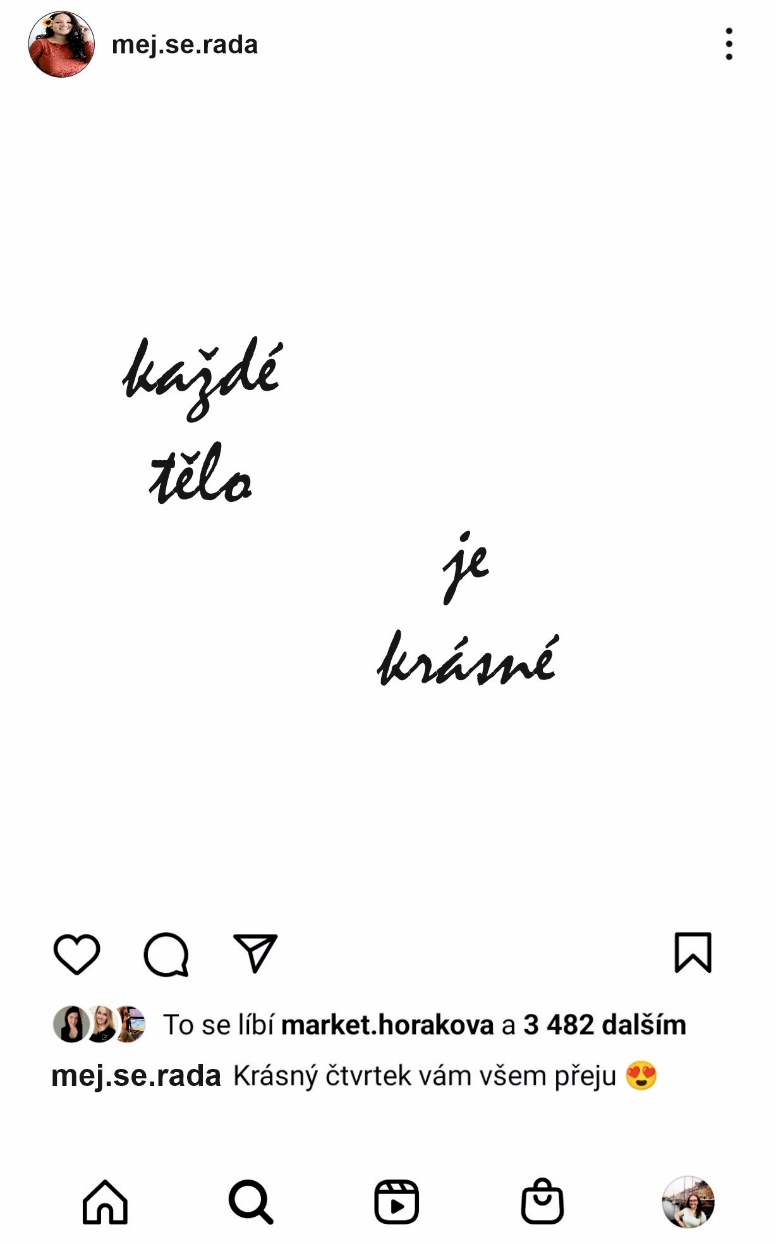 | 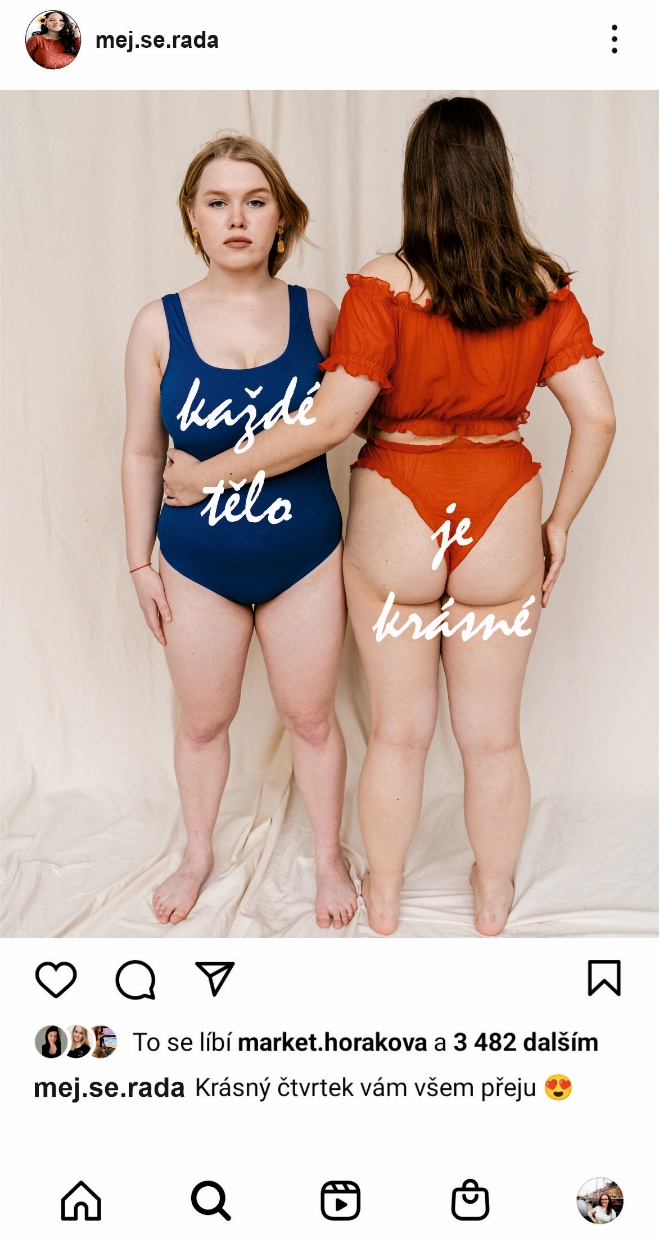 |
| 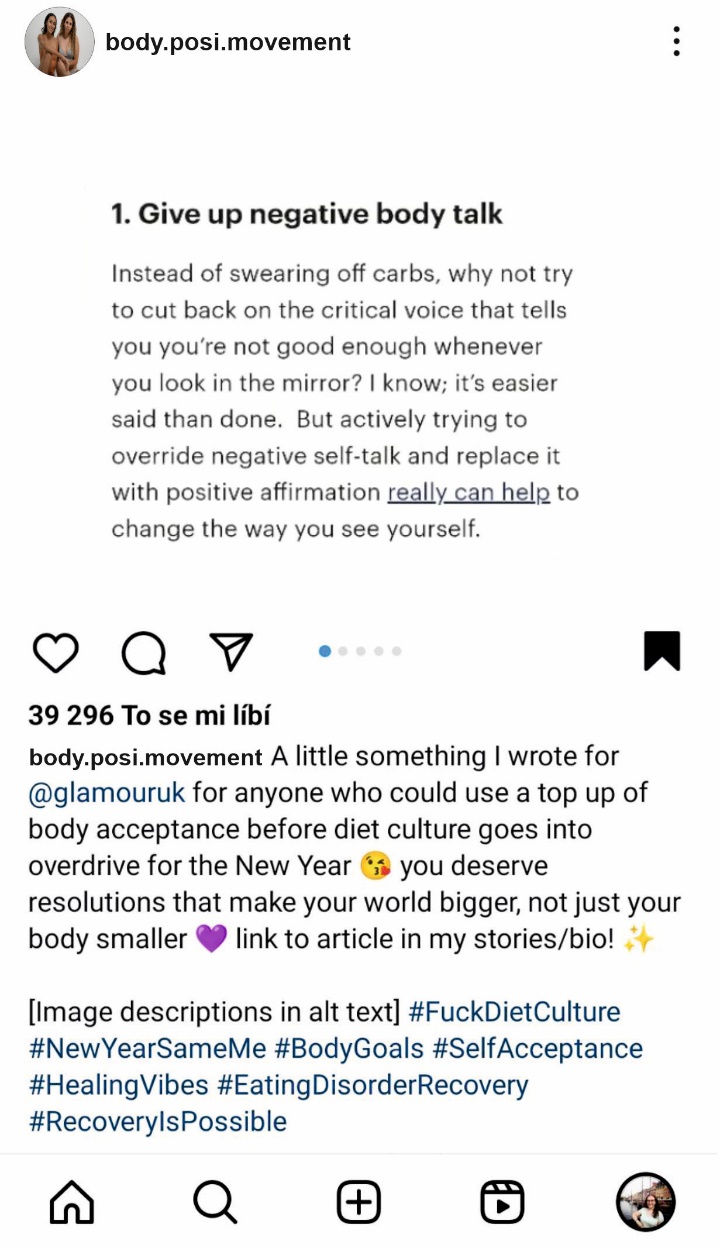 | 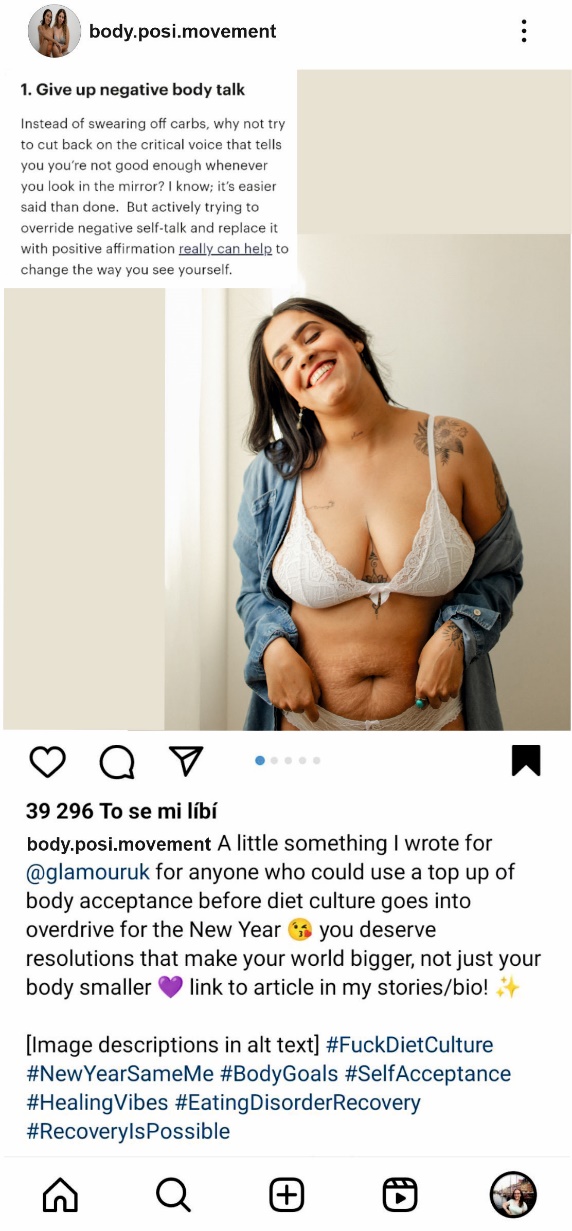 |
| 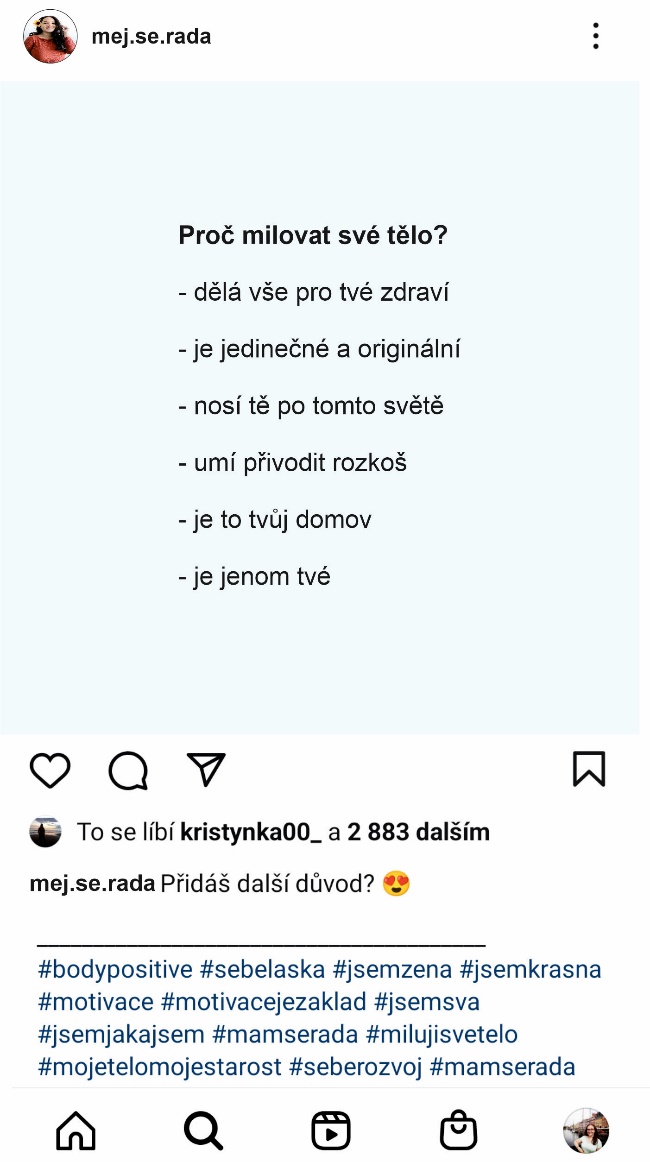 | 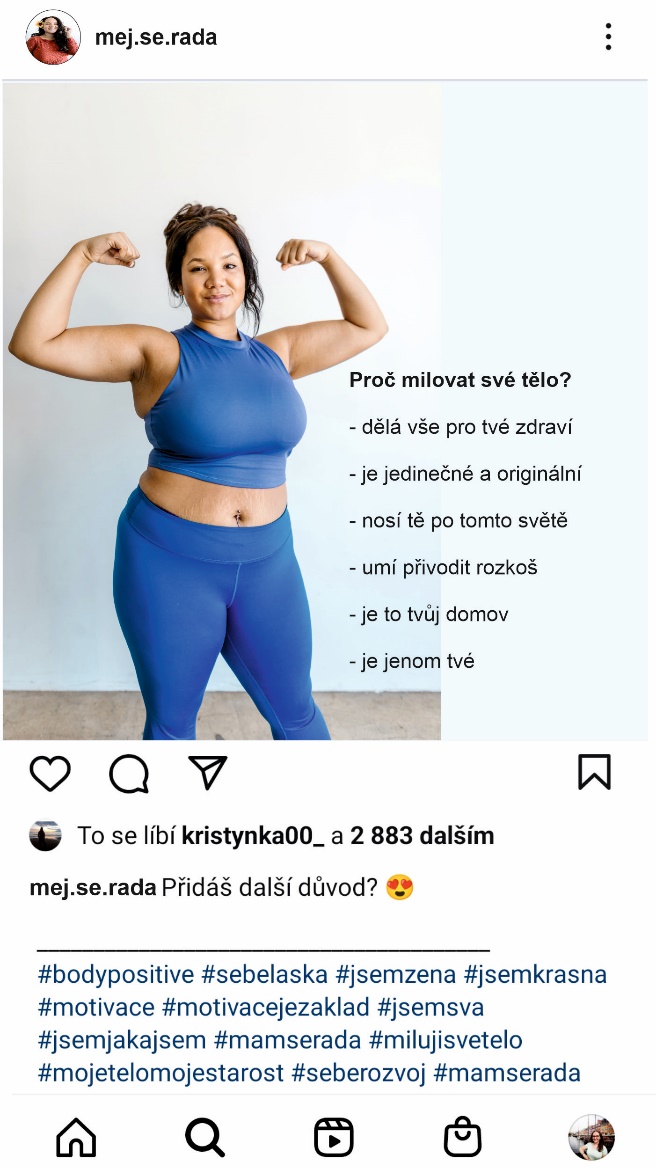 |
| 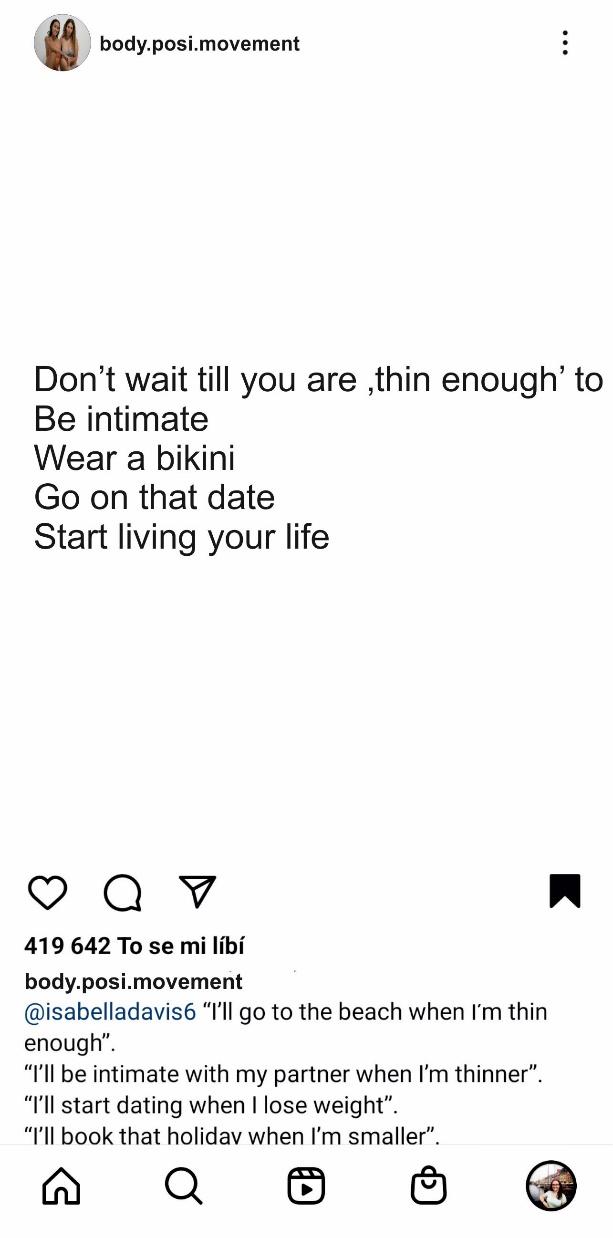 | 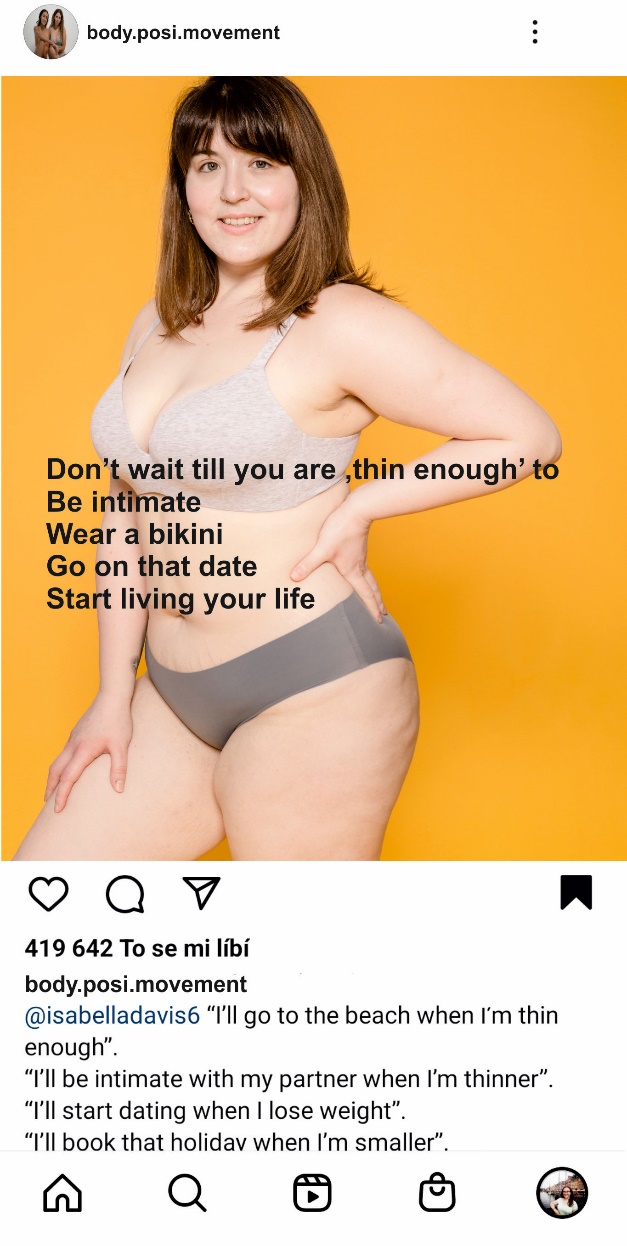 |
| 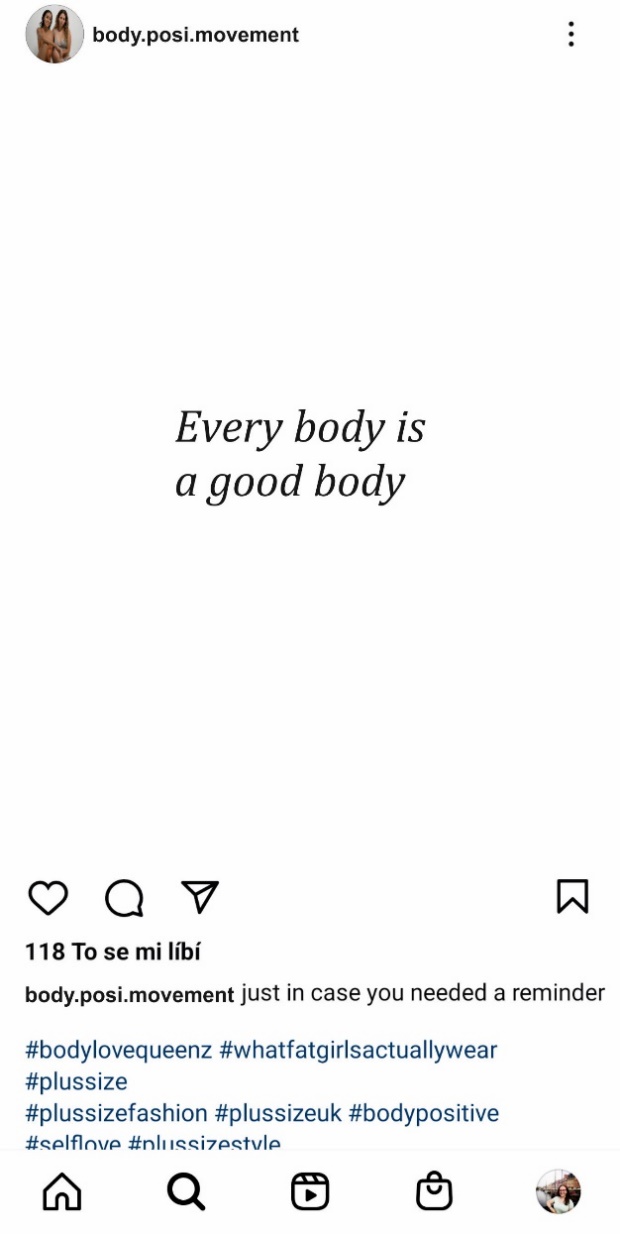 | 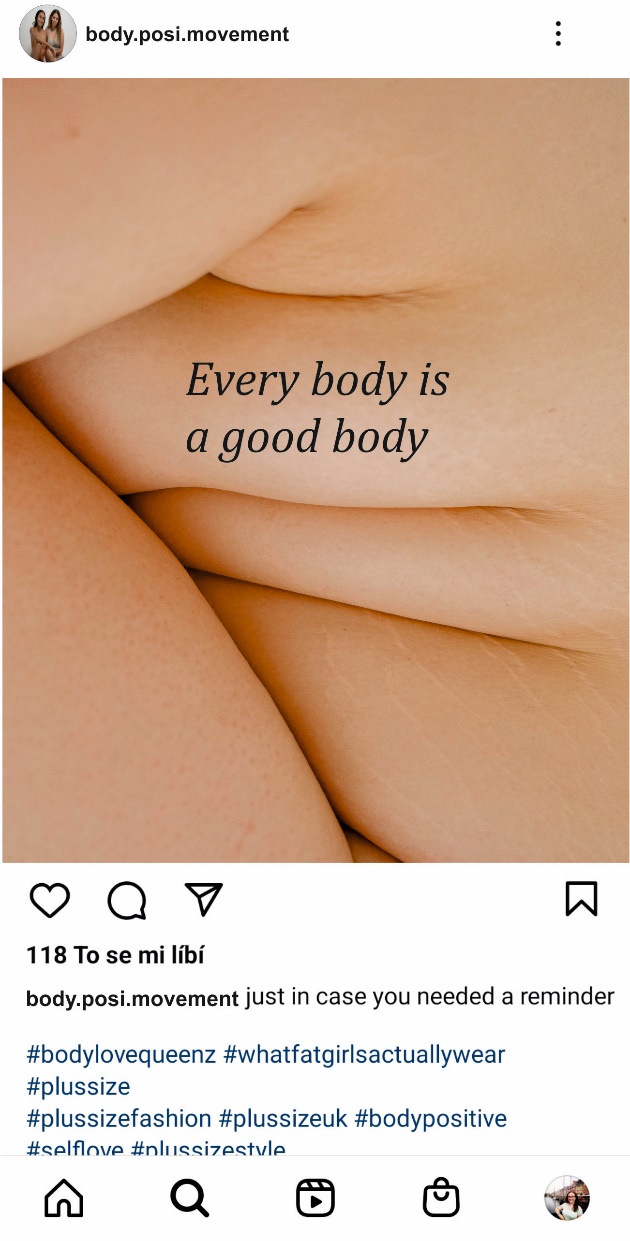 |
| 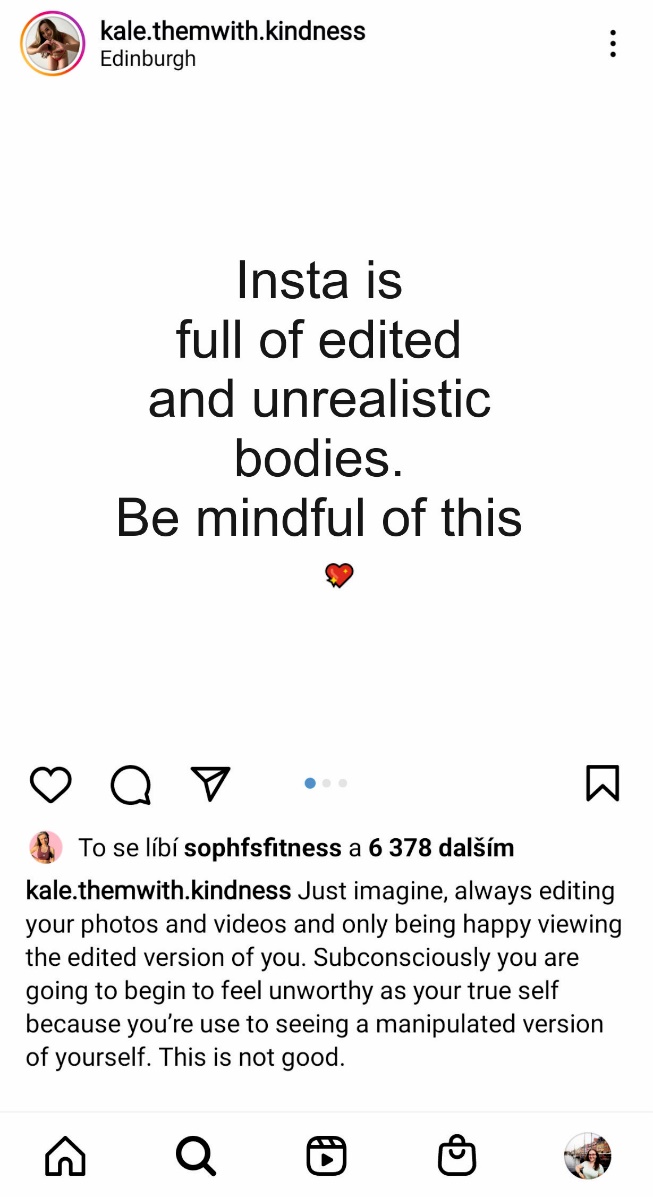 | 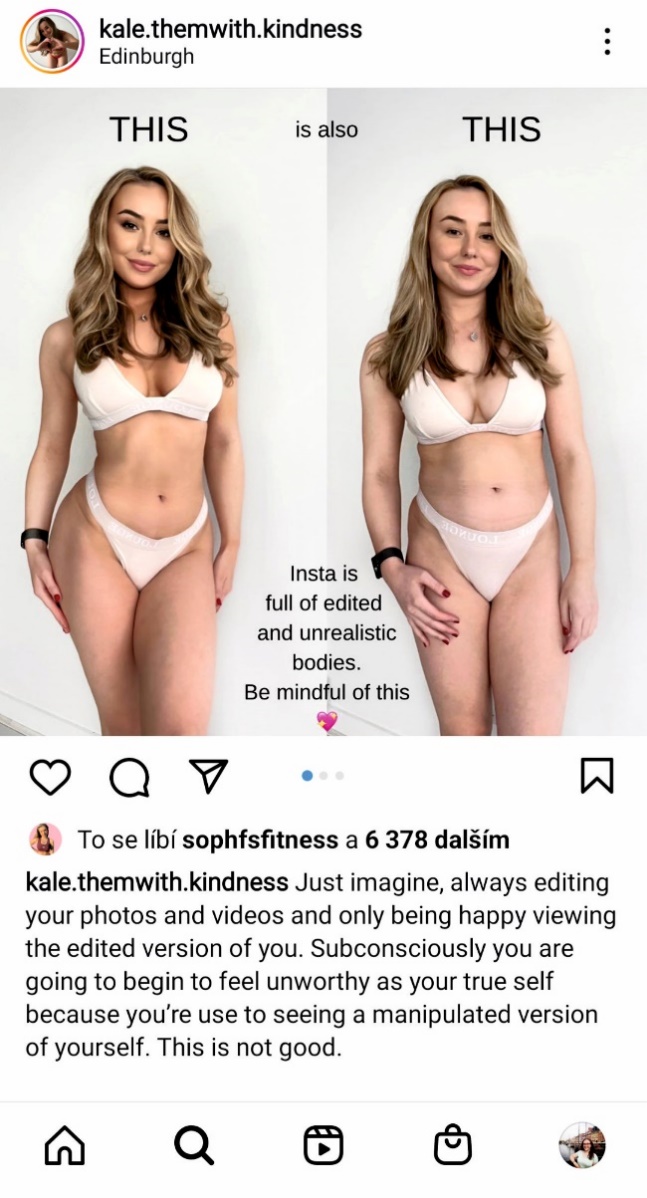 |

**Supplementary Appendix F. Measurement of State Self-objectification**

**Ten Statements Test**

*Original version*

Describe yourself using ten sentences beginning with "I am ".

*Translation*

Popište se prosím pomocí 10 vět, které začínají slovy "já jsem".

1. Já jsem
2. Já jsem
3. Já jsem
4. Já jsem
5. Já jsem
6. Já jsem
7. Já jsem
8. Já jsem
9. Já jsem
10. Já jsem

**Body Surveillance Scale**

*Original version*

1. I rarely think about how I look.
2. I think it is more important that my clothes are comfortable than whether they look good on me.
3. I think more about how my body feels than how my body looks.
4. I rarely compare how I look with how other people look.
5. During the day, I think about how I look many times.
6. I often worry about whether the clothes I am wearing make me look good.
7. I rarely worry about how I look to other people.
8. I am more concerned with what my body can do than how it looks.

Reverse coded items: 1, 2, 3, 4, 7, 8

*Translation and adding “right now”*

1. Právě teď nepřemýšlím o tom, jak vypadám.
2. Právě teď považuji za důležitější, aby bylo mé oblečení pohodlné, než abych v něm dobře vypadala.
3. Právě teď více přemýšlím o tom, jak se cítím ve svém těle, než jak mé tělo vypadá.
4. Právě teď srovnávám svůj vzhled se vzhledem ostatních.^[[2]](#footnote-2)^
5. Právě teď přemýšlím, jak vypadám.
6. Právě teď se obávám, jestli v oblečení, které mám na sobě, vypadám dobře.
7. Právě teď mě trápí, jak vypadám pro ostatní.^[[3]](#footnote-3)^
8. Právě teď se spíše zajímám o to, jak je mé tělo funkční, než jak vypadá.

Reverse coded items: 1, 2, 3, 8

**Supplementary Appendix G. Measurement of attitudes toward body positivity**

**Instructions:**

Please express your level of agreement with the following statements.

If you are not familiar with the term "body positivity," consider it based on this definition: Body positivity accounts reject unrealistic body ideals and encourage women to accept and love their bodies of any shape and size. Body positivity posts on Instagram typically feature women who proudly showcase their unique bodies along with quotes and texts about body acceptance.

**Statements:**

1. Watching body positivity posts can lead to a more positive perception of my body.

2. Body positivity posts evoke positive emotions in me.

3. I follow body positivity accounts on some social media platforms.

**Supplementary Appendix H. Debriefing**

Explanation of the Research Objective

This research examines the impact of body positivity posts on the body image of the viewer, specifically on the level of self-objectification that viewing these posts may increase. We define self-objectification as viewing oneself as an object to be evaluated. Exploring this area is considered important because self-objectification has several negative consequences, particularly on one's perception of their own body and body satisfaction. As long as body positivity posts continue to increase self-objectification, they will not succeed in helping their followers accept and love their bodies.

In the questionnaire, you saw either posts that displayed only text or posts that displayed both text and photographs of women. This is because participants were randomly divided into two groups, with each group viewing one type of post. After viewing the posts, you completed a questionnaire to measure the degree of your self-objectification. The goal of this study is to determine whether there was a difference in the current self-objectification between the two groups and the different posts. I hypothesize that a slightly greater increase will be observed in the group that viewed the posts with photos of women because the female body has the potential to be objectified. This may be due to the fact that media often portrays women as objects whose bodies tell us everything about them (including who they are and how they behave).

If, after learning about the research details, you decide that you do not want to participate, please write a message in this column.

1. This definition was added after a misunderstanding in the pilot study. [↑](#footnote-ref-1)
2. We have changed the original negative formulation to a positive one in order to make the item more understandable. The original item was reverse-coded, but it is not after the change. [↑](#footnote-ref-2)
3. Same change as the item above. [↑](#footnote-ref-3)
